# Predictive Factors for Major Complications and Urological Cancer Diagnosis in Older Adults (≥80 Years) Admitted to the Emergency Department for Hematuria

**DOI:** 10.3390/jcm13102874

**Published:** 2024-05-13

**Authors:** Mauro Ragonese, Daniele Fettucciari, Luigi Carbone, Filippo Gavi, Marco Montesi, Eros Scarciglia, Pierluigi Russo, Domenico Maria Sanesi, Filippo Marino, Nazario Foschi, Francesco Pinto, Francesco Franceschi, Marco Racioppi, Emilio Sacco, Marcello Covino

**Affiliations:** 1 Department of Urology, Fondazione Policlinico Universitario A. Gemelli IRCCS, 00136 Rome, Italyfilippo.marino01@icatt.it (F.M.);; 2 Facoltà di Medicina e Chirurgia, Università Cattolica del Sacro Cuore, 00136 Rome, Italyemilio.sacco@policlinicogemelli.it (E.S.); marcello.covino@policlinicogemelli.it (M.C.); 3 Department of Emergency Medicine, Ospedale Fatebenefratelli Isola Tiberina, Gemelli Isola, 00186 Rome, Italy; 4 Emergency Medicine, Fondazione Policlinico Universitario A. Gemelli IRCCS, 00136 Rome, Italy; 5 Department of Urology, Ospedale Fatebenefratelli Isola Tiberina, Gemelli Isola, 00186 Rome, Italy

**Keywords:** visible hematuria, emergency department, urological neoplasm, bladder cancer, sepsis, urological urgency, older adults

## Abstract

**Background**: Gross Hematuria is a relevant cause of admission to the emergency department in the general population and particularly in older adults (≥80 years). This specific urological symptom is often underestimated and usually associated with benign conditions such as urinary infections or poor hydration. Nevertheless, hematuria could lead to severe acute complications or be the first symptom of urological cancers. **Methods**: We retrospectively analyzed clinical data from 1169 patients aged ≥80 years consecutively admitted to the emergency department for hematuria. The primary endpoint of the study was to identify risk factors for major complications, and the secondary endpoint was to analyze risk factors for urological cancer diagnosis. The median age was 85 years (IQR 82–88 years), and 908 (77%) were males. Among them, 449 (38.4%) had a past medical history of urological neoplasm (kidney, ureter, bladder, prostate, or urethral cancer). **Results:** Overall, 87 patients (7.4%) had major complications (patient death, septic shock, and admission to the intensive care unit). Worse vital signs at admission, fever, and confusion (*p* < 0.001, OR 18.0 IC 95% [5.5–58.7]; *p* = 0.015, OR 2.0 IC 95% [1.1–3.5]; *p* = <0.001, OR 4.2 IC 95% [1.9–3.5], respectively), as well as lower hemoglobin values and higher Charlson comorbidity index (*p* < 0.001, OR 0.8 IC 95% [0.7–0.9]), *p* = 0.002, OR = 1.2 [1.1–1.3]) were independent predictive factors for major complications. The multivariate analysis identified as risk factors for diagnosis of urological cancer older age, male sex and higher comorbidity (OR 1.05 IC95% [1–1.09]; OR 2.19 IC95% [1.42–3.39] and OR 1.11 IC95% [1.2–1.2], respectively); interestingly the presence of indwelling vesical catheter (IVC) (OR 0.44 IC95% [0.24–0.82]) resulted as an independent factor for absence of urological cancers. **Conclusions:** Hematuria is a frequent symptom in older adults admitted to the emergency department. While this is often associated with benign conditions, there are some risk factors for major complications and for urological cancer that must be taken into account to identify the patients who need further evaluation or prompt hospital admission.

## 1. Introduction

The elderly population (aged ≥ 80 years) has increased over the last decades at a steady pace, having a major effect on the cost of healthcare services [1,2]. Projections show that by 2050, 22% of the worldwide population will be over 60 years old [3]. According to the US Census Bureau middle-mortality series projections, people aged 85 years and older are expected to exceed 12 million by 2040 in the United States [4]. In this setting, emergency departments provide a fundamental service to older adults to access both acute and long-term healthcare services [5]. Every day, many patients admitted to the emergency department (ED) for urological issues suffer from visible hematuria (VH), accounting for around 20% of urological consults [6]. Hematuria is defined as the presence of red blood cells in the urine: microscopic hematuria when detected by the examination of urinary sediment; gross hematuria when visible to the naked eye [6]. VH can result in a huge concern for the patient, causing anxiety and distress [7], especially when presenting for the first time.

There are many possible causes, both benign and life-threatening [8], and this symptom could be the typical presenting sign of bladder cancer (BC), which accounts for the 10th most commonly diagnosed tumor when both genders are considered and for the 6th most commonly diagnosed tumor in males [9]. Nowadays, as a result of a delayed urological consult, imaging, and cystoscopy, women usually get a diagnosis later than men, presenting with more advanced-stage bladder cancer [10,11].

In patients presenting with VH, the ED evaluation should distinguish patients with a risk for progression to life-threatening conditions such as hemorrhagic shock or sepsis and should possibly rule out the presence of unknown urological malignancies. This is particularly true in older patients since the presence of several comorbidities and overall frailty reduces the risk/benefit ratio of invasive diagnostic procedures. At the same time, the presence of coexistent comorbidity may lead to an overlook of VH.

Given these premises, this study aims to define what are the main risk factors for urological cancer diagnosis and for the occurrence of major complications in older adults (aged ≥ 80 years) presenting with VH at ED.

## 2. Materials and Methods

### 2.1. Study Design and Inclusion Criteria

This is a retrospective, single-center, cohort study conducted in the ED of a tertiary care university hospital (Fondazione Policlinico Universitario “Agostino Gemelli” IRCCS of Rome) with an average attendance of about 75,000 patients per year, of which more than 87% are adults. Study reporting was performed according to the “Enhancing the QUAlity and Transparency Of health Research” (EQUATOR) guidelines using the “Strengthening The Reporting of OBservational studies in Epidemiology” (STROBE) checklist.

We included all consecutive patients aged 80 years or more admitted to our ED for gross hematuria from January 2018 to December 2024. Patients with incomplete or inconsistent clinical records were excluded from the final analysis. We also excluded from the analysis patients with gross hematuria secondary to major trauma.

### 2.2. Study Variables and Definitions

The clinical records of the eligible patients were retrospectively collected from a prospectively maintained database and identified using the International Classification of Disease, 9th Revision, Clinical Modification (ICD-9-CM) codes, as follows: 599.7 (Hematuria) either as a primary diagnosis or as a secondary diagnosis.

The following demographic and clinical data were collected: age, gender, comorbidities (coronary artery disease, chronic heart failure, cerebrovascular disease, dementia, cirrhosis, diabetes, chronic obstructive pulmonary disease, chronic kidney disease, malignancy, known urological malignancy, presence of metastasis, presence of an indwelling vesical catheter (IVC) or a percutaneous nephrostomy (PCN), HIV) including the Charlson Comorbidity Index (CCI), laboratory parameters (white blood cells (WBC) count, hemoglobin, platelets, serum creatinine, serum glucose, red cell distribution width (RDW); level of urgency (minor-urgent, urgent, emergency) according to the National Early Warning Score (NEWS2 [12,13]); clinical presentation at ED admission (fever, abdominal pain, chest pain, asthenia, syncope); type of treatment (non-surgical management, surgical treatment either endoscopic procedure or percutaneous/open surgery); disease complications at ED admission or appeared during ED staying (septic shock or admission to ICU) and post-management outcomes (in-hospital death and length of hospital stay (LOS)). LOS was calculated from the time of ED admission to discharge or death. Mortality was defined as any death occurring within 30 days after ED admission.

### 2.3. Management of Visible Hematuria

According to the local protocol in the ED, all the VH patients had a clinical and diagnostic work-up as follows:

- Comprehensive medical and surgical history and physical examination;
- Blood and urine tests;
- Radiological imaging. Kidney–ureter–bladder (KUB) ultrasound (US) was the first line imaging investigation used to assess the patient. If the KUB was negative, and if there was high clinical suspicion, a CT scan was obtained (with or without intravenous contrast, based on the differential diagnosis and renal function);
- Placement of a three-way silicone catheter and, after manual bladder irrigation to remove clots, continuous bladder irrigation.

When a clinical suspect of bladder cancer was present and/or radiological imaging was inconclusive, a cystoscopy was requested and performed either during the ED stay or delayed to an ambulatory setting.

Patients with solved VH were discharged to an outpatient follow-up. The patients with persistent hematuria, those with signs of acute infection, those with severe hemorrhage, and those with coexistent acute medical conditions were admitted to the hospital ward.

### 2.4. Study Endpoints

The primary endpoint of the study is to define predictive factors for major complications (MC) and urological oncological neo-diagnosis in patients admitted to ED with VH.

Major complications were defined as death, septic shock, or need for admission to the ICU.

As secondary endpoints, we evaluated:

Need for hospital admission.

### 2.5. Statistical Analysis

Categorical variables are presented as absolute numbers and percentages; continuous variables are presented as median (interquartile range). Categorical variables were compared using the Chi-square test or Fisher exact test as appropriate. Continuous variables were compared using the Mann–Whitney U test. Significant factors at univariate analysis were entered into a Logistic regression model to identify independent risk predictors for the defined outcomes. A *p*-value of 0.05 was regarded as significant in all the analyses.

Data were analyzed using IBM SPSS statistics for Windows, Version 25 (IBM Corp. Armonk, NY, USA) and MedCalc Statistical Software version 19.2.1 (MedCalc Software Ltd., Ostend, Belgium).

### 2.6. Sample Size Post-Hoc Calculations

Considering the need for at least 10 events for each degree of liberty of the multivariate model, the study cohort is adequate for the multivariate parameter estimation in the case of prediction of major complications and the presence of urological malignancy in naïve patients. Since the reduced number of deaths in our cohort, the sample size is underpowered for the multivariate estimation of the factors associated with in-hospital death.

## 3. Results

### 3.1. Baseline Characteristics

In the study period, 1169 patients aged ≥ 80 years were admitted to our ED with visible hematuria and included in the study cohort.

The median age was 85 years (IQR 82–88 years), and 908 (77%) were males. A past medical history of urological neoplasm (kidney, ureter, bladder, prostate, or urethral cancer) was found in 449 (38.4%), of which 401 (89.3%) were male (Table 1).

Patients with known urological malignancy were more frequently male, generally with a higher derangement of vital parameters evaluated with the NEWS2 score, and were often admitted to the ED for fever (Table 1). These patients also had significantly lower WBC (white blood cell) and platelet count (*p* = 0.004 and *p* = 0.021, respectively). Not unexpectedly, these patients had a higher number of comorbidities (Table 1).

### 3.2. Clinical Outcomes

A total of 348 (34.8%) patients presented with VH due to a urological neoplasm; 87 (7.4%) had major complications, of which 2 (0.17%) were admitted to the ICU.

Overall, 439 (37.5%) patients were admitted to the hospital from the Emergency Department (ED). Hospital admission was significantly higher in patients who had a known urological tumor rather than those who did not (46.8% vs. 31.8%, respectively, *p* < 0.001), and, as expected, even the need for surgery was significantly higher in patients with previously known urological cancers.

### 3.3. Predictors of Major Complications

Factors associated with MC in univariate and multivariate analyses are shown in Table 2.

In the univariate analysis, more than one factor was shown to be associated with a higher risk of MC: a higher NEWS2 score, fever, asthenia, confusion, and the use of anticoagulants (*p* < 0.001; *p* = 0.34; *p* = 0.002; *p* < 0.001; *p* = 0.04, respectively) as ED presentation parameters; a lower hemoglobin level (*p* < 0.001) as laboratory finding; a higher CCI, previous deep vein thrombosis, transient ischemic attack, dementia, chronic pulmonary disease, chronic heart failure and the presence of metastasis at ED admission time (*p* < 0.001; *p* < 0.001; *p* = 0.022; *p* = 0.015; *p* = 0.013; *p* < 0.001; *p* < 0.001) as relevant past medical history factors.

The multivariate analysis confirmed a higher NEWS2 score, fever, and confusion to be independent variables associated with higher odds of MC (*p* < 0.001, OR 18.0 CI 95% [5.5–58.7]; *p* = 0.015, OR 2.0 CI 95% [1.1–3.5]; *p* < 0.001, OR 4.2 CI 95% [1.9–3.5], respectively). A lower hemoglobin value resulted as a predictive factor for MC risk (*p* < 0.001, OR 0.8 CI 95% [0.7–0.9]). A higher CCI results as a negative predictive factor for major complications (*p* = 0.002, 1.2 [1.1–1.3]). However, specific comorbidities such as deep vein thrombosis, transient ischemic attack, dementia, chronic pulmonary disease, chronic renal failure, and the presence of tumor metastasis were significant only in the univariate analysis.

### 3.4. Predictor of Urological Malignancies

Among 720 patients admitted to our ED for VH without a positive past medical history of urological tumor, 168 (23%) were diagnosed with a urological neoplasm (Table 3). In particular, bladder cancer 130 (77.4%); prostate cancer 11 (6.55%); kidney cancer 20 (11.9%); ureteral cancer 6 (3.57%).

At the univariate analysis age, male sex, a lower hemoglobin value, higher creatinine blood levels, higher CCI, cirrhosis, and the presence of another neoplasm (*p* = 0.018; *p* = 0.001; *p* = 0.015; *p* < 0.001; *p* = 0.003; *p* = 0.003; *p* = 0.033; *p* < 0.001; *p* = 0.003; *p* < 0.001 and *p* < 0.001, respectively) were associated with a higher risk of presenting urological cancer.

At the multivariate analysis, age, male sex, and higher CCI (OR 1.05 CI 95% [1–1.09]; OR 2.19 CI 95% [1.42–3.39] and OR 1.11 CI 95% [1.2–1.2], respectively) resulted as independent factors associated with a higher risk of an underlying urological malignancy. On the other hand, the presence of an indwelling vesical catheter (IVC) (OR 0.44 CI 95% [0.24–0.82]) is an independent factor for reduced odds of urological cancer diagnosis.

## 4. Discussion

Major findings of the present study are several clinical factors that could be associated with a higher risk of major complications or with a previously unknown urological malignancy in older adults (≥80 years) accessing the ED for gross hematuria. As previously shown, in the ED setting, the goal of treatment for gross hematuria is summarized as “RESP” (Resuscitation, Ensuring adequate urinary drainage, Safe discharge, Prompt follow-up) [14].

Resuscitation of the patient is the first step when cardiovascular failure is evident, aiming for volume replacement and correction of any coagulopathy or anemia. Ensure adequate urinary drainage is even mandatory when there is urinary retention and bladder obstruction. The use of a silicone catheter with the possibility of bladder irrigation is, therefore, essential. Safe discharge is the next step when there is no need for further immediate investigations, with Prompt follow-up of the patient and referral to the specialist as subsequent steps in the management.

The first step is to evaluate hemodynamic stability and discern the need for surgical intervention or the risk of major complications [8]. This evaluation is often only based on physician experience, the patient’s past medical history, and physical examinations [15]. Potential diagnoses may include infections, kidney stones, benign prostatic hyperplasia, nephrological problems, trauma, anticoagulant drugs, recent procedures, and tumors [14,16,17,18,19,20,21,22]. Not always red to brown color urine implies blood in the urine. Older adults often take multiple medications, which may change urine color [23] (e.g., levodopa, pyridium, riboflavin, doxorubicin, blackberries, paprika, nitrofurantoin, and sulfonamides). This condition is defined as “pseudo hematuria” [24] and a negative urine dipstick test can confirm the absence of hematuria. In agreement with the literature [6,7,16,17,23,24], the workup for hematuria should always start with a comprehensive medical and surgical history and physical examination.

Until proven otherwise, however, a tumor should be suspected in all cases of visible hematuria [17]. Even in the case of non-visible hematuria, a urological tumor should be excluded; therefore, further investigation is needed. An elective computed tomography (CT) scan may help identify malignancies, having a 98% positive predictive value and a 76% negative predictive value in genitourinary tract cancer detection [25,26]. Urine tests, blood tests, and radiological imaging (US, CT) are the milestones that should always be run at ED presentations, especially when bladder cancer is suspected [27].

Cystoscopy (flexible or rigid) is the investigation of choice for the lower urinary tract as it can be performed quickly, allowing diagnostic discernment if imaging investigation shows equivocal or abnormal results [28,29].

Considering the high prevalence of hematuria in older patients and the wide spectrum of benign conditions that could be associated with this symptom, it could be of great relevance to identify in the ED setting the clinical factors associated with poor prognosis. The present study revealed that a higher risk of major complications (sepsis, admission to ICU, or death) is associated with confusion, fever, a higher CCI score, and a lower hemoglobin level. The latter both reflects the severity of the bleeding or the worsening of a prior anemic condition (which affects almost 17% of older adults [30]). Similarly, the augmented frailty of these patients, as reflected by their higher number of comorbidities, is most likely the cause of their poorer outcomes, in agreement with previous research [5,31,32]. Among these studies, Samaras et al. particularly stress the fact older adults are admitted to ED more frequently and for longer than younger patients, having a 2.5 to 4.6 times higher risk for hospitalization and a fivefold higher admission rate to an ICU [31].

Contrary to common sense and previous findings, we found no statistical association in the multivariate analysis between the use of anticoagulants or aspirin and the occurrence of MC in our cohort. This is in contrast with Wallis et al. [33], who found the use of antithrombotic medications to be significantly associated with higher rates of hematuria-related complications compared with the non-use of these medications. However, in this latter study, MC was defined as ICU admission, hospitalization, and the need for urological procedures to control the bleeding.

Among the 1169 patients admitted, as expected, males were prevalent due to the high incidence of prostate-related VH and the well-known higher prevalence of urological malignancies in male patients.

Among the 720 patients without previous history of urological malignancy, 168 (23.3%) were diagnosed with malignancies in the study cohort. Older age, higher CCI, and male sex were identified as predictive factors for the diagnosis. It is, therefore, mandatory to focus the attention on these patients who might have a late diagnosis of urological cancers due to their comorbidities and their fragility. The results in this study population ≥80 years matched the epidemiologic data of Burger et al. in the general population [34], confirming both male gender and age as risk factors for urothelial cancer diagnosis. Furthermore, our results highlight the importance of urological consult when VH is present in both genders: even if bladder cancer is less prevalent in females, the underestimation of hematuria in women, due to the differential diagnosis with benign conditions such as urinary infections, entails a higher risk of a late diagnosis, resulting in more advanced-stage bladder cancer.

As expected, there was a statistically significant difference in terms of need for surgery and length of stay (*p* < 0.001 and *p* = 0.003, respectively) between patients diagnosed with cancer rather than those with other diagnoses.

Interestingly, IVC resulted as a negative predictive factor, as expected, considering the higher odds of benign conditions for hematuria in these patients, such as urinary infections.

To the best of our knowledge, this study is the first to evaluate VH in patients ≥80 years, and further research is needed to help define the best work-up for these patients, as it would possibly help reduce complication rates and make faster diagnoses. Also, it would be of great interest to identify a VH stratification method able to stratify the population into risk categories, analogous to the one made by the American Urology Association (AUA) for microhematuria [6].

### Study Limitations

It must be noted that this study has several limitations that should be acknowledged.

Firstly, as this study is confined to patients of a single center, our findings may not be generalizable to the wider community. Secondly, as this is a referral center for urological neoplasms in Italy, the number of patients diagnosed with a urological tumor may not reflect the prevalence in the general population.

## 5. Conclusions

Gross hematuria is a common presenting condition at ED in patients ≥80 years old and should be investigated as it is often associated with a urological malignancy and the risk for major complications.

Taking into account these risk factors could allow better risk stratification of these patients in the ED, to identify those who need close clinical monitoring, prompt further investigations, and hospital admission. A higher NEWS2 score, fever, confusion and a higher CCI score are the red flags clinicians should be aware of when treating VH.

To our knowledge, this is the first study that identifies risk factors for oncological diagnosis in patients ≥80 years old admitted for hematuria to the ED. Future research should aim to develop a clinical risk score that could be applied to facilitate the management of these patients.

## Figures and Tables

**Table 1:** Demographical and clinical characteristics of patients ≥80 years of age presenting with acute visible hematuria in the Emergency Department. About half of the evaluated patients had no prior history of urological disease.

	All Patients N 1169	Patients with Known Urological Malignancy N 449	Controls N 720	*p* Value
*Age*	85 (82–88)	85 (82–88)	85 (82–88)	0.421
*Sex (male)*	908 (77.7%)	401 (89.3%)	507 (70.4%)	*<0.001*
***ED Presentation***				
*Fever*	234 (20%)	72 (16.0%)	162 (22.5%)	*0.007*
*Abdominal Pain*	209 (17.9%)	77 (17.1%)	132 (18.3%)	0.607
*Chest Pain*	17 (1.5%)	5 (1.1%)	12 (1.7%)	0.442
*Asthenia*	56 (4.8%)	22 (4.9%)	34 (4.7%)	0.890
*Syncope*	20 (1.7%)	8 (1.8%)	12 (1.7%)	0.883
***Triage code***				
*Emergency*	16 (1.4%)	3 (0.7%)	13 (1.8%)	
*Urgency*	427 (36.5%)	188 (41.9%)	239 (33.2%)	0.004
*Minor Urgency*	726 (62.1%)	258 (57.5%)	468 (65.0%)	
***Laboratory Values***				
*Hemoglobin*	11.6 (10–13)	11.4 (9.5–12.9)	11.7 (10.2–13.1)	0.059
*WBC*	8.3 (6.6–10.7)	7.5 (5.7–9.1)	8.6 (6.9–11.2)	*0.004*
*Platelets*	218 (167–289)	192 (137–270)	231 (170–306)	*0.021*
*RDW*	15 (13.9–16.7)	14.9 (13.8–16.7)	15 (13.9–16.8)	0.865
*Creatinine*	1.17 (0.86–1.69)	1.18 (0.84–1.89)	1.17 (0.90–1.65)	0.928
*Glucose*	119 (101–133)	127 (106–139)	114 (101–128)	0.093
***Urological History***				
*Cancer*	449 (38.4%)	449 (38.4%)	0	*<0.001*
*PCN*	68 (5.8%)	39 (8.7%)	29 (4.0%)	*<0.001*
*IVC*	145 (12.4%)	49 (10.9%)	96 (13.3%)	0.222
***Comorbidities***				
*Charlson Index*	6 (5–8)	6 (5–8)	6 (4–8)	*0.002*
*IM*	328 (28.0%)	132 (29.4%)	196 (27.2%)	0.420
*HF*	292 (25.0%)	111 (24.7%)	181 (25.1%)	0.873
*TIA*	60 (5.1%)	15 (3.3%)	45 (6.3%)	*0.028*
*Dementia*	95 (8.1%)	21 (4.7%)	74 (10.3%)	*0.001*
*COPD*	103 (8.8%)	44 (9.8%)	59 (8.2%)	0.346
*Diabetes*	161 (13.8%)	71 (15.8%)	90 (12.5%)	0.110
*CRF*	313 (26.8%)	120 (26.7%)	193 (26.8%)	0.976
*HIV*	2 (0.2%)	1 (0.2%)	1 (0.2%)	0.736
**Outcomes**				
Admission from ED	439 (37.5%)	210 (46.8%)	229 (31.8%)	*<0.001*
Open Surgery	42 (3.6%)	24 (5.3%)	18 (2.5%)	*0.011*
Endoscopic Surgery	91 (7.8%)	50 (11.1%)	41 (5.7%)	*0.001*
Death	38 (3.2%)	15 (3.3%)	23 (3.2%)	0.888
CMC	87 (7.4%)	32 (7.1%)	55 (7.6%)	0.751
LoS	0.9 (0.3–3.1)	0.8 (0.3–2.7)	1.0 (0.3–4.1)	*0.005*

Abbreviations: CCI—Charlson Comorbidity Index; COPD—Chronic Pulmonary Disease; CRF—Chronic Renal Failure; DM—Diabetes Mellitus; ED—Emergency Department; HF—Heart Failure; IVC—Indwelling Vesical Catheter; LoS—Length of Stay; MI—Myocardial Infarction; PCN—Percutaneous Nephrostomy; RDW—Red Cell Distribution Width; TIA—Transient Ischemic Attack; UTI—Urinary Tract Infection; WBC White Blood Cells. CMC—cumulative major complications.

**Table 2:** Factors associated with the occurrence of major complications (septic shock, admission to intensive care unit, and death) in the study cohort.

	Patients with Major Complications N 87	Controls N 1082	*p* Value	Odds Ratio (95% Confidence Interval)	Multiv. *p* Value
**Age**	85 (82–88)	85 (82–88)	0.934		
**Sex (male)**	73 (83.9%)	836 (77.2%)	0.148		
ED Presentation					
**Triage Code**					
** Emergency**	9 (10.3%)	7 (0.6%)		18.0 [5.5–58.7]	<0.001
** Urgency**	52 (59.8%)	376 (34.7%)	<0.001	2.7 [1.6–4.7]	<0.001
** Minor Urgency**	26 (29.9%)	700 (64.6%)		*Reference*	<0.001
**Fever**	25 (28.7%)	209 (19.3%)	0.034	2.0 [1.1–3.5]	*0.015*
**Abdominal Pain**	20 (23.0%)	189 (17.5%)	0.195		
**Asthenia**	10 (11.5%)	46 (4.2%)	0.002	1.1 [0.4–2.5]	0.883
**Syncope**	1 (1.1%)	19 (1.8%)	0.675		
**Thoracic Pain**	2 (2.3%)	15 (1.4%)	0.493		
**Confusion**	15 (17.2%)	26 (2.4%)	<0.001	4.2 [1.9–3.5]	*<0.001*
**Urological history**	44 (50.6%)	523 (48.3%)	0.682		
**Known Urological Tumor**	32 (36.8%)	417 (38.5%)	0.751		
**Percutaneous Nephrostomy**	3 (3.4%)	65 (6.0%)	0.327		
**IVC**	8 (9.2%)	137 (12.7%)	0.347		
**Use of anticoagulants**	11 (12.6%)	73 (6.7%)	0.040	2.0 [0.9–4.5]	0.077
**Use of Aspirin**	34 (39.1%)	431 (39.8%)	0.895		
Laboratory Values					
**Hemoglobin**	9.8 (8.7–12.2)	11.7 (10.2–13.1)	<0.001	0.8 [0.7–0.9]	*<0.001*
**WBC**	8.86 (6.9–19)	8.2 (6.58–11.8)	0.300		
**Platelets**	254 (207–278)	215 (164–296)	0.332		
**RDW**	14.7 (13.4–17.1)	15 (13.9–16.7)	0.555		
**Cr**	1.2 (0.85–1.91)	1.17 (0.88–1.66)	0.864		
**Glucose**	114 (99–162)	120 (101–133)	0.838		
Comorbidities					
**CCI**	7 (6–9)	6 (4–8)	<0.001	1.2 [1.1–1.3]	*0.002*
**MI**	19 (21.8%)	309 (28.5%)	0.181		
**HF**	24 (27.6%)	268%)	0.556		
**DVT**	18 (20.7%)	40 (3.7%)	<0.001		
**TIA**	9 (10.3%)	51 (4.7%)	0.022		
**Dementia**	13 (14.9%)	82 (7.6%)	0.015		
**COPD**	14 (16.1%)	89 (8.2%)	0.013		
**Hepatopathy**	0 (0.0%)	12 (1.1%)	0.324		
**Cirrhosis**	1 (1.1%)	9 (0.8%)	0.756		
**DM**	17 (19.5%)	144 (13.3%)	0.104		
**CRF**	39 (44.8%)	274 (25.3%)	<0.001		
**Other Neoplasm**	32 (36.8%)	395 (36.5%)	0.954		
**Metastasis**	7 (8.0%)	11 (1%)	<0.001		
**HIV**	0 (0%)	2 (0.2%)	0.688		
Outcomes					
**Admission from ED**	79 (90.8%)	360 (33.2%)	<0.001		
**Open Surgery**	5 (5.7%)	37 (3.4%)	0.261		
**Endoscopic Surgery**	15 (17.2%)	76 (7.0%)	0.001		
**LoS**	11.3 (5.1–20.2)	0.8 (0.2–2.2)	<0.001		

Abbreviations: CCI—Charlson Comorbidity Index; COPD—Chronic Pulmonary Disease; CRF—Chronic Renal Failure; DM—Diabetes Mellitus; DVT—Deep Vein Thrombosis; ED—Emergency Department; HF—Heart Failure; IVC—Indwelling Vesical Catheter; LOS—Length of Stay; MI—Myocardial Infarction; RDW—Red Cell Distribution Width; TIA—Transient Ischemic Attack; WBC White Blood Cells.

**Table 3:** Factors associated with a diagnosis of urological malignancy in the 720 patients without a previous neoplastic history and acute hematuria in the Emergency Department.

	Urological Malignancy N 168	Other Diagnoses N 552	*p* Value	Odds Ratio (95% Confidence Interval)	Multiv. *p* Value
**Age**	86 (83.3–89)	85 (82–88)	0.018	1.05 [1.00–1.09]	0.033
**Sex (male)**	136 (81%)	372 (67.3%)	0.001	2.19 [1.42–3.39]	<0.001
ED Presentation					
**Triage Code**					
** Emergency**	3 (1.8%)	10 (1.8%)			
** Urgency**	50 (29.8%)	190 (34.4%)	0.538		
** Minor Urgency**	168 (68.5%)	353 (63.8%)			
**Fever**	30 (17.9%)	132 (23.9%)	0.102		
**Abdominal Pain**	29 (17.3%)	103 (18.6%)	0.689		
**Hypotension**	8 (4.8%)	29 (5.2%)	0.804		
**Syncope**	3 (1.8%)	9 (1.6%)	0.888		
**Chest Pain**	4 (2.4%)	8 (1.4%)	0.407		
**Confusion**	4 (2.4%)	28 (5.1%)	0.139		
**Urological History (any)**	23 (13.7%)	95 (17.2%)	0.284		
**Nephrostomy**	9 (5.4%)	20 (3.6%)	0.315		
**IVC**	13 (7.7%)	83 (15%)	0.015	0.44 [0.24–0.82]	0.009
**Use of anticoagulants**	7 (4.2%)	46 (8.3%)	0.071		
**Use of Aspirin**	73 (43.5%)	234 (42.3%)	0.794		
Laboratory Values					
**Hemoglobin**	10.8 (9.6–12.3)	11.9 (10.3–13.2)	<0.001	0.79 [0.57–1.09]	0.162
**WBC**	8.6 (6.2–11.8)	9.15 (7.17–11.15)	0.349		
**Platelets**	252 (168–326)	225 (170–290)	0.710		
**RDW**	14.9 (13.6–16.9)	15.3 (13.9–16.8)	0.714		
**Cr**	1.38 (1.19–1.87)	1.04 (0.76–1.41)	0.003	1.23 [0.62–2.44]	0.116
**Glucose**	148 (102–128)	109 (97–130)	0.576		
Comorbidities					
**CCI**	6 (5–8)	6 (4–7)	0.003	1.11 [1.02–1.20]	0.015
**MI**	49 (29.2%)	147 (26.6%)	0.510		
**HF**	37 (22%)	144 (26%)	0.293		
**TIA**	6 (3.6%)	39 (7.1%)	0.102		
**Dementia**	12 (7.1%)	62 (11.2%)	0.128		
**COPD**	16 (9.5%)	43 (7.8%)	0.469		
**Cirrhosis**	4 (2.4%)	3 (0.5%)	0.033		
**DM**	23 (13.7%)	67 (12.1%)	0.589		
**CRF**	41 (24.4%)	152 (27.3%)	0.429		
**Other Neoplasm**	86 (51.2%)	114 (20.6%)	<0.001		
**HIV**	1 (0.6%)	0 (0%)	0.069		
Outcomes					
**LoS**	1.25 (0.30–5.41)	0.75 (0.25–2.25)	0.003		
**Sepsis**	3 (1.8%)	19 (3.4%)	0.276		
**Open Surgery**	12 (7.1%)	6 (1.1%)	<0.001		
**Endoscopic Treatment**	23 (13.7%)	18 (3.3%)	<0.001		
**Major Complications**	9 (5.4%)	46 (8.3%)	0.205		
**Hospital admission**	64 (38.1%)	165 (29.8%)	0.044		

Abbreviations: CCI—Charlson Comorbidity Index; COPD—Chronic Pulmonary Disease; CRF—Chronic Renal Failure; DM—Diabetes Mellitus; ED—Emergency Department; HF—Heart Failure; IVC—Indwelling Vesical Catheter; LOS—Length of Stay; MI—Myocardial Infarction; RDW—Red Cell Distribution Width; TIA—Transient Ischemic Attack; WBC White Blood Cells.

## Data Availability

The raw data supporting the conclusions of this article will be made available by the authors on request.
